# Pathophysiological and neurobehavioral characteristics of a propionic acid-mediated autism-like rat model

**DOI:** 10.1371/journal.pone.0192925

**Published:** 2018-02-15

**Authors:** Jeonghyun Choi, Seunghoon Lee, Jinyoung Won, Yunho Jin, Yunkyung Hong, Tai-Young Hur, Joo-Heon Kim, Sang-Rae Lee, Yonggeun Hong

**Affiliations:** 1 Department of Rehabilitation Science, Graduate School of Inje University, Gimhae, Korea; 2 Biohealth Products Research Center (BPRC), Inje University, Gimhae, Korea; 3 Ubiquitous Healthcare & Anti-aging Research Center (u-HARC), Inje University, Gimhae, Korea; 4 Department of Physical Therapy, College of Healthcare Medical Science & Engineering, Inje University, Gimhae, Korea; 5 Animal Biotechnology Division, National Institute of Animal Science, Wanju, Korea; 6 Institute of Animal Medicine, College of Veterinary Medicine, Gyeongsang National University, Jinju, Korea; 7 National Primate Research Center (NPRC), Korea Research Institute of Bioscience and Biotechnology (KRIBB), Ochang, Korea; Radboud University Medical Centre, NETHERLANDS

## Abstract

Autism spectrum disorder (ASD) is induced by complex hereditary and environmental factors. However, the mechanisms of ASD development are poorly understood. The purpose of this study was to identify standard indicators of this condition by comparing clinical, pathophysiological, and neurobehavioral features in an autism-like animal model. A total of 22 male Sprague-Dawley rats were randomly divided into control and 500 mg/kg propionic acid (PPA)-treated groups. Rats were subjected to behavioral tests, gene expression analyses, and histological analyses to detect pathophysiological and neurobehavioral alterations. Exploratory activity and non-aggressive behavior were significantly reduced in PPA-treated rats, whereas enhanced aggressive behavior during adjacent interactions was observed on day 14 after PPA administration. To evaluate gene expression after PPA administration, we analyzed hippocampal tissue using reverse transcription PCR. Glial fibrillary acidic protein was augmented in the PPA-treated group on day 14 after appearance of ASD-like behaviors by PPA administration, whereas octamer-binding transcription factor 4 expression was significantly decreased in the PPA-treated group. Histological evaluation revealed significantly reduced diameter and layer thickness of granule cells in PPA-treated rats compared with control rats. We conclude that PPA administration induced abnormal neural cell organization, which may have led to autism-like neurobehaviors, including increased aggressive behavior, reduced exploratory activity, and isolative and passive behaviors.

## Introduction

Autism spectrum disorder (ASD) is characterized by social deficits, repetitive and restricted behaviors, and alteration of brain development [1]. Several studies have reported an interrelation between brain development and autistic neurobehavior. Additionally, changes in hippocampal structures are related to ASD behavior [2]. Generally, neurogenesis in the dentate gyrus is related to long-term potentiation in memory function [3] and the development of neuropsychiatric disorders [4] after birth. In addition, approximately 15% of granule cells, which make up the dentate gyrus, are produced during embryogenesis [5]. Therefore, the dentate gyrus is an important region involved in ASD development. The estimated prevalence of ASD is about 2.64% in South Korea [6], and the disorder is 3–4 fold more prevalent in males than in females. Autism is usually diagnosed before 36 months of age. The disorder is highly heritable and neuropsychiatric, as evidenced by the higher concordance rates in monozygotic (82–92%) compared with dizygotic (1–10%) twins [7]. ASD is a neurodevelopmental disorder with a complex etiology that is not fully understood.

Genetic and environmental factors affect the development of ASD [8]. Several studies have shown that ASD pathogenesis is affected by genetic, metabolic, immunological, gastrointestinal (GI), environmental, and behavioral factors [9–13]. A previous study suggested that metabolites, including short-chain fatty acids, produced via fermentation of foods by microbes, may affect the systemic immune system, hormone secretion, and even the central nervous system (CNS) of patients with ASD [8, 14]. Many research groups have explored whether gut microbiome metabolites induce ASD. In addition, several novel animal models of ASD have been developed by modulating gut metabolite levels in various ways [15, 16].

Propionic acid (PPA) is a gut metabolite, and its generation is related to clostridials, and others; microbial changes in response to antibiotics and pro/prebiotics have been reported. In human patients with ASD, exposure to excessive antibiotics results in altered microbial biogeography, which affects dysbiosis and systemic inflammation and leads to the pathophysiology of GI diseases and ASD [17]. ASD-like rat models have been generated using various routes of PPA administration: subcutaneous (500 mg/kg), intragastric gavage (250 mg/kg), intraperitoneal (250 mg/kg), and intracerebroventricular (4 uL of 0.26 M PPA, pH 7.5). Following PPA administration, model rats exhibited elevated levels of microglia (CD68 positive) and neurotoxic cytokines, including interleukin (IL)-6, tumor necrosis factor (TNF)-α, and interferon-γ, as well as abnormal neurobehaviors, such as repetitive and impaired social interactions [18–21]. Additionally, PPA and its derivative, nitropropionic acid, affect hippocampus-related diseases such as ASD and Huntington’s disease [8]. In a previous study, PPA-treated rats exhibited increased expression of oxidative markers (oxidized lipid and proteins) and decreased activities of glutathione (GSH) and glutathione peroxidase compared with the phosphate-buffered saline (PBS)-treated group [15, 16]. Ossenkopp et al. [22] reported that administering PPA (500 mg/kg) intraperitoneally to rats weighing 200–300 g produced both taste avoidance and avoidance behaviors because of its irritant effects. Therefore, in rats, systemic PPA administration alters behaviors regardless of age.

Valproic acid (VPA), a well-known risk factor for ASD development during the prenatal phase, has similar structural and pharmacological properties as PPA does [23]. Several studies reported that intraperitoneal (500 mg/kg) and subcutaneous (400 mg/kg) VPA injections induced many behavioral changes, including social interaction deficits, reductions in acoustic pre-pulse inhibition and attention, and increased anxiety-like behavior [24–26]. A recent study reported that long-term (9–12 days) exposure to VPA (100 mg/kg) in utero resulted in an increased number of neocortical neurons in rat pups postnatally [27]. Various methods have been suggested to generate ASD-like animal models, including administration of PPA or VPA. Both PPA and VPA have similar effects including inhibition of histone deacetylase, altering carnitine activity and mitochondrial metabolism [23]. However, VPA has several side effects, including hepatic steatosis [28], hepatotoxicity, hemorrhagic pancreatitis, encephalopathy, and metabolic disorders such as obesity [29].

Clinical approaches have been used to identify the pathophysiological mechanisms of ASD. Thus, basic research using a standard animal model and neurobehavioral protocols is needed. The purpose of this study was to identify standard indicators of ASD by comparing clinical, pathophysiological, and neurobehavioral features following a change in brain structure using an ASD-like animal model induced by PPA administration.

## Materials and methods

### Experimental animals and procedure

**Fig 1:**
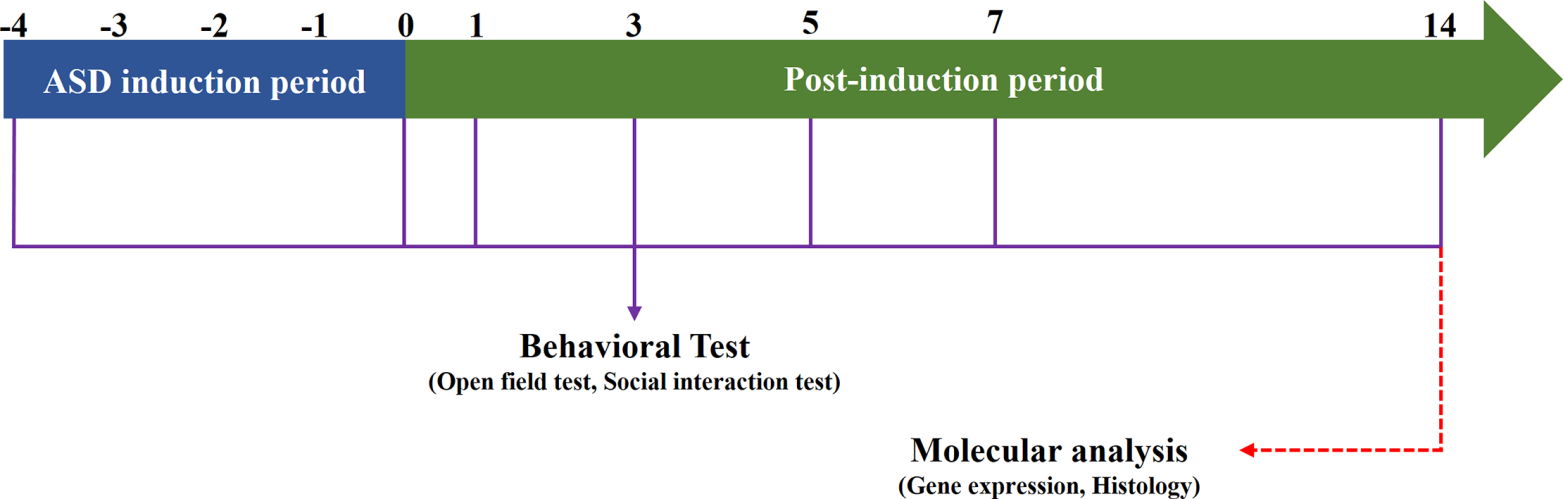
Schematic representation of experimental design.

A total of 22 healthy male Sprague–Dawley rats weighing 80–100 g, were obtained from Daehan BioLink (Hoychang Science, Daegu, Korea) and used in all studies. All experimental procedures for evaluating ASD development were performed on 3-week-old animals. The rats were randomly divided into either control or PPA-treated (ASD) groups. Animals were allowed access to standard rodent chow (Hyochang Science) and tap water *ad libitum*. All animal study procedures were approved by the Ethics Committee for Animal Care and Use of Inje University (Approval No. 2014–21), which is certified by the Korean Association of Accreditation of Laboratory Animal Care. All rats were housed two per cage under controlled environmental conditions (22 ± 1°C) and an established light:dark photoperiod (12:12 hr; lights on: 07:00). The experimental procedures are illustrated in Fig 1.

### PPA administration

Sodium propionate (PPA, Sigma-Aldrich. St. Louis, MO, USA) was dissolved in 0.1 M PBS and administered subcutaneously at a dose of 500 mg/kg (250 mg/mL, 0.26 M, pH 7.4) once a day for five consecutive days. This dose was selected based on previous studies [14, 20]. Physiologically, PPA is a weak acid that readily crosses the blood brain barrier or the gut-blood barrier. Increased PPA levels in organs lead to intracellular acidification [21] and induce systemic inflammation via upregulation of pro-inflammatory cytokine concentrations in the CNS. The pH of PPA used was 7.4, which is close to that of the physiological buffer, and a solution with this pH easily crosses lipid barriers. Therefore, pH 7.4 PPA directly affects the CNS and shows systemic irritant effects [15, 22]. Rats in the control group were injected with saline. In this study, post-induction day (PID) represents the day after five times consecutive PPA administration into the rats for five days. In addition, body weight and ASD phenotypes were counted every single day from PID 0 (day 0) in turn. Immune system activation caused by postnatal PPA administration results in abnormal behaviors and augments the susceptibility of later systemic insults. Additionally, repeated stimulation of immune activation by PPA administration may change the biogeography of gut microbes, which is related to the production of aversive metabolites [30, 31].

### Neurobehavioral testing

#### Social interaction test

The social interaction test was conducted in a 120 × 120 × 60 cm black acrylic arena illuminated by a 40 W red lamp. Age- and treatment-matched pairs of rats with equivalent body weights but from different cages were placed together in the arena for 20 min. This test measured the following five categories of behavior: (1) following/chasing: one rat following the other within a distance of 2 body lengths; (2) anogenital interactions; (3) adjacent interactions: play-fighting, climbing over/under, and adjacent lying; (4) head-to-head interactions; and (5) total social interaction: time spent engaging in all behaviors in all of the above categories. The apparatus was cleaned with 50% ethanol between test sessions [32].

#### Open filed test (olfactory discrimination)

Animals were randomly assigned to the test order and placed in a white acrylic square-form open field arena (100 × 100 × 40 cm). As reported previously [25], this arena was placed under strong illumination (200 lux). The arena was divided into 25 squares (each square was 20 × 20 cm): 9 central and 16 peripheral squares. Each rat was placed in the center of the open field and allowed to explore the environment for 1 min. After that, the number of squares that the rat crossed was recorded by a video camera for 5 min and divided into the number of outer squares (those adjacent to the walls) crossed (outer locomotion) and the number of inner squares crossed (inner locomotion). The arena was cleaned with 50% ethanol between tests.

### RNA preparation and reverse transcription polymerase chain reaction (RT-PCR)

The rats were anesthetized using mixed gasses containing 3% isoflurane with O_2_ and N_2_O. The brain tissues were removed, and the hippocampal region was isolated. The tissues were homogenized with 1 mL of Tri-reagent (Sigma-Aldrich, St. Louis, MO, USA) to prepare total RNA. The RNA was reverse transcribed with oligo(dT) 12–18 using reverse transcriptase #18064–014 (Invitrogen, Carlsbad, CA, USA) and this reaction mixture served as a template for the PCR. To identify gene transcription, a reaction mixture (50 uL) for PCR was prepared using 2.0 uL of cDNA synthesis mixture, 40 nM dNTPs, 10 pM of sense and antisense primer, and 1.25 U of GoTaq® DNA polymerase (Promega, Madison, WI, USA). PCR was performed with denaturation at 95°C for 30 sec, annealing at 60°C for 1 min, and extension at 72°C for 1 min in each cycle, followed by a final 10 min extension at 72°C using C1000 Touch^TM^ Thermal Cycler (BioRad, Hercules, CA, USA). The following primers were used. Primer sequences: microtubule-associated protein 2 (*MAP2*) as a neuronal marker, CAA AGA GAA GGT GGC AAA GC (F), GTG GGC AAG GGA TTT CTA CA (R); glial fibrillary acidic protein (*GFAP*) as an astrocyte marker, TGG CCA CCA GTA ACA TGC AA (F), CAG TTG GCG GCG ATA GTC AT (R); octamer-binding transcription factor 4 (*OCT4*) as a stem cell marker, GAG GGA TGG CAT ACT GTG GAC (F), GGT GTA CCC CAA GGT GAT CC (R); and tumor necrosis factor (*TNF*)*-α* as a pro-inflammatory cytokine marker, CTA CTG AAC TTC GGG GTG ATC (F), CTT GTC CCT TGA AGA GAA CCT G (R).

### Histological evaluation

All rats were transcardially perfused with 0.1 M PBS (pH 7.4), and subsequently fixed with 4% neutral-buffered paraformaldehyde (pH 7.4) for histological analyses. Brain tissues were removed from the 4% neutral-buffered paraformaldehyde solution and stored at 4°C for 2 hr in fixative solution (post-fixation). Tissues were incubated in a gradient of sucrose solution (15% and 30%) for 1 day to achieve a cryoprotective effect. Next, tissues were transferred to an embedding mold fabricated from aluminum foil filled with Tissue-Tek® OCT compound (Sakura Finetek, Torrance, CA, USA). The mold was rapidly submerged in isopentane cooled with liquid nitrogen. After the material was frozen, the block was wrapped in cellophane and aluminum foil and stored at -70°C. Cryosectioning was performed at the optimal temperature (brain: -18°C to -20°C) using a cryostat microtome (Microm HM525; MICROM International GmbH, Walldorf, Germany). The block face was trimmed to create a round shape, with the long axis oriented vertically. This orientation facilitated removal of the sections from the knife edge and minimized damage caused by handling the tissue. After trimming, the tissues were cut carefully (slice thickness: 10 μm), and a small camel-hair brush was used to guide the section off the block face and transfer it to a gelatin-coated slide. The section was left to dry on the slide at room temperature for 15 min. Tissue sections were subjected to cresyl violet acetate (Nissl) staining, serially dehydrated with ethanol solutions, cleared with xylene, and mounted with toluidine solution (Fisher Scientific Co., Fair Lawn, NJ, USA). Each specimen was analyzed using an Olympus microscope digital camera (Olympus, Tokyo, Japan) connected to a computer. To evaluate the thickness of the granule cell layer (GCL) and cell diameter, Image-Pro Plus software (Media Cybernetics, Inc., Rockville, MD, USA) was used. In this software, we adjusted the scale bar to pixel units.

### Immunostaining

Rats were anesthetized as described above and perfused transcardially with 4% paraformaldehyde in 0.1 M phosphate buffer, pH 7.4, for 15 min. Post-fixation was performed overnight in 4% paraformaldehyde. For fluorescence immunostaining, non-specific labeling was blocked with 0.1% bovine serum albumin in 0.1% Triton X-100/PBS for 60 min. The following primary antibody was used and incubated with the tissue overnight at 4°C: mouse monoclonal anti-GFAP (1:300, Cell Signaling Technology, Danvers, MA, USA). The slides were then incubated with goat anti-mouse conjugated to rhodamine (1:500, Molecular Probes, Eugene, OR, USA) secondary antibody for 60 min. Each specimen was analyzed using an Olympus BX51 microscope and DP70 digital camera (Olympus, Tokyo, Japan) and connected to a computer using Image-Pro Plus software (Media Cybernetic Inc., Rockville, MD, USA). A three dimensional (3D) plot type graph was used to determine the number of positive cells.

### Statistical analysis

Data were collected from repeated experiments and are presented as the mean ± standard deviation. Student`s *t*-tests were used for the statistical analysis, and differences were considered significant when *p < 0.05 and **p < 0.01. All analyses were performed using SPSS software (SPSS ver. 20.0; SPSS Inc., Chicago, IL, USA).

## Results

### Physiological changes after PPA administration

**Fig 2:**
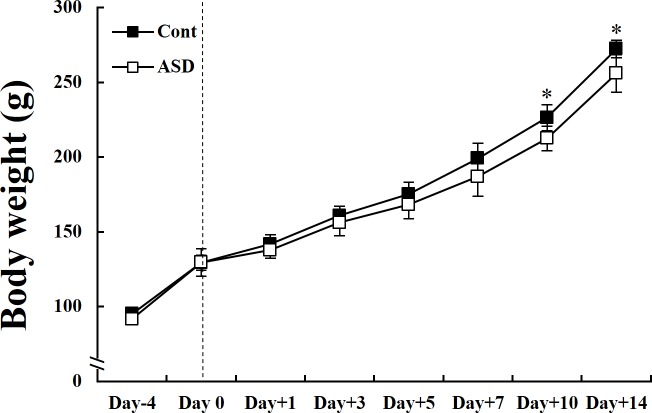
Changes in body weight between control and PPA-treated rats. Alteration in body weight between control and PPA-treated groups during whole PID period (days 0 to 14). PPA-treated rats weighed significantly less than control rats during the late PID period (days 10 and 14). Data are presented as the mean ± standard deviation (SD). Cont, control rats; ASD, PPA-treated rats. ^*^p < 0.05: *vs*. Cont.

We assessed alterations in body weight between control and PPA-treated groups during the whole period of PID (days 0 to 14). PPA-treated rats did not show altered body weight compared with control rats during the early PID period (days 0 to 3). However, there were significant differences in body weight during the late PID period (days 10 and 14) (*p < 0.05; Fig 2). We also measured food consumption to determine the correlation between body weight and food consumption. However, there were no differences between food consumption and weight loss (S1 Fig).

### Open field exploratory activity following PPA administration

**Fig 3:**
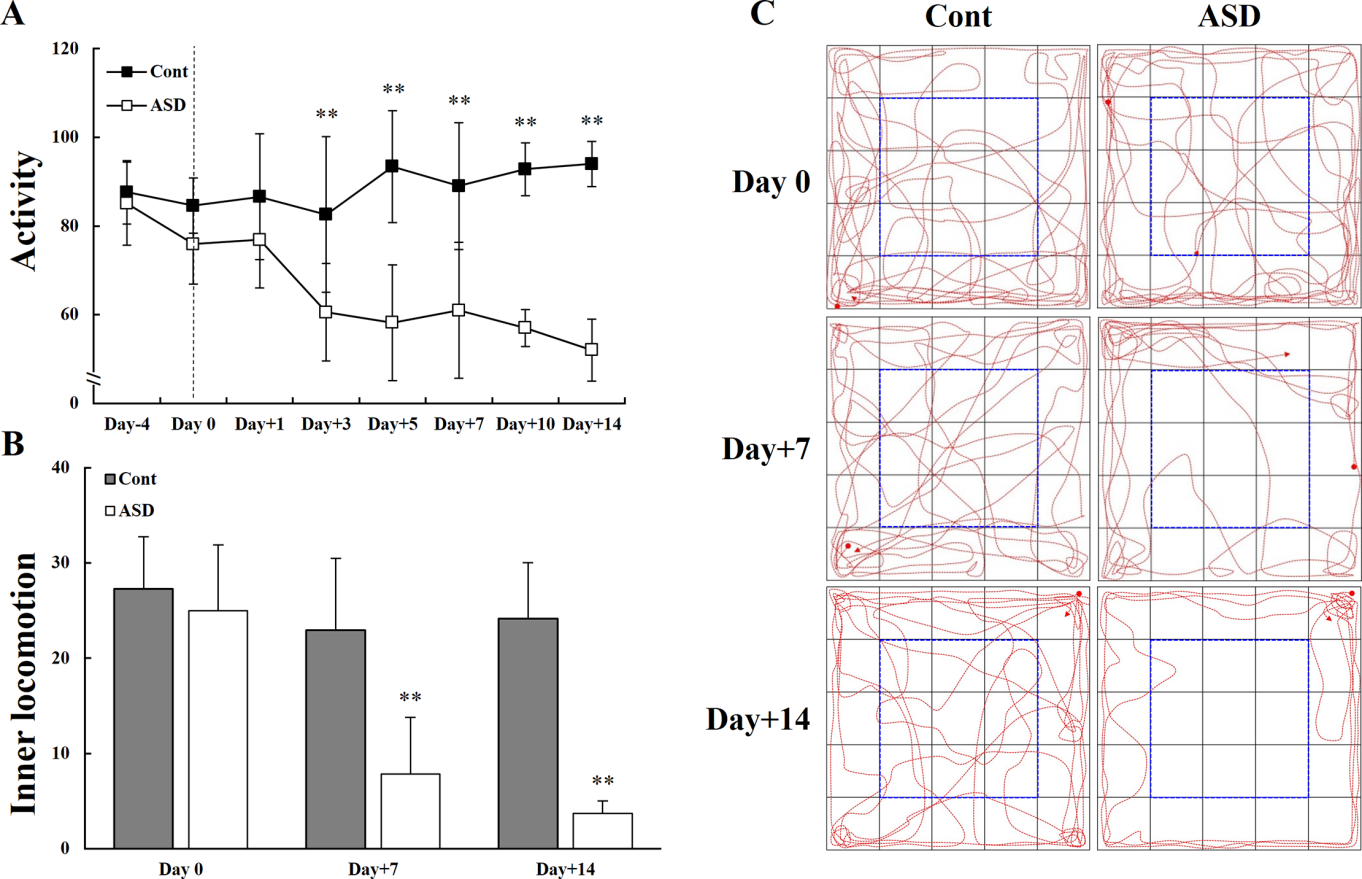
Comparison of exploratory activity in a non-social environment between control and PPA-treated groups. PPA-treated rats showed significantly reduced exploratory activity (A) and inner-square exploratory activity (B) during the whole PID period (days 0 to 14). (C) The locomotor activity of the control and PPA-treated rats was observed using a video tracking system. Data are presented as the mean ± SD. Cont, control rats; ASD, PPA-treated rats. ^*^p < 0.01: *vs*. Cont.

We measured exploratory activity in a non-social environment between early (days 0 to 3) and late PID period (days 7 to 14). PPA-treated rats showed slightly reduced exploratory activity in a non-social environment during the early PID period. However, they exhibited a significant attenuation in both total activity from PID 3 and inner-square exploratory locomotion on PID 7 and 14 (**p < 0.01; Fig 3A and 3B). Indeed, PPA-treated rats showed a progressive reduction in their inner-square exploratory activity during the late PID period (Fig 3C); they also urinated and defecated more than did the control rats during the static period.

### Social behavior after PPA administration

**Fig 4:**
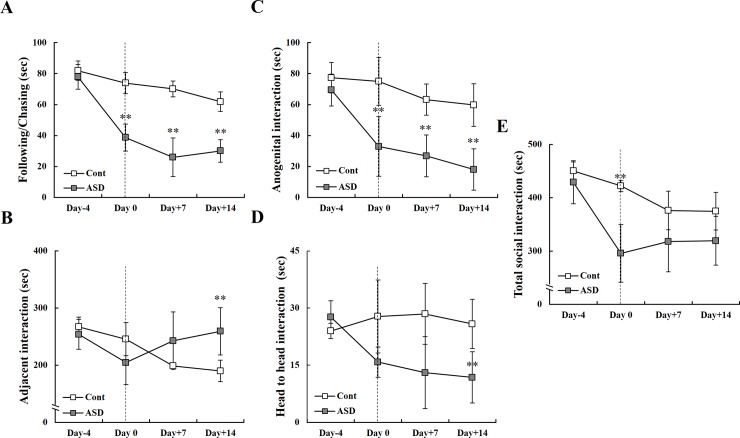
Alterations in social interaction between early and late post-induction day following PPA administration. (A, C, D) PPA-treated rats showed significantly reduced non-aggressive behavior, but (B) increased aggressive behavior during the late PID period (days 7 and 14). (E) All social interactions between the control and PPA-treated rats are shown. Data are presented as the mean ± SD. Cont, control rats; ASD, PPA-treated rats. ^*^p < 0.01: *vs*. Cont.

We evaluated four categories of social behavior between control and the PPA-treated groups during the whole PID period (days 0 to 14): following and chasing (Fig 4A), adjacent interaction (Fig 4B), anogenital interaction (Fig 4C), and head-to-head interaction (Fig 4D, S1 Video). PPA-treated rats showed significantly attenuated non-aggressive behaviors, including following and chasing, anogenital interactions, and head-to-head interactions (Fig 4A, 4C and 4D). However, we also observed significantly increased aggressive behavior during adjacent interactions on PID 14 (Fig 4B). The total number of social interactions decreased significantly on PID 0, indicating consistent ASD-like behaviors (**p < 0.01; Fig 4E).

### Gene expression following PPA administration

**Fig 5:**
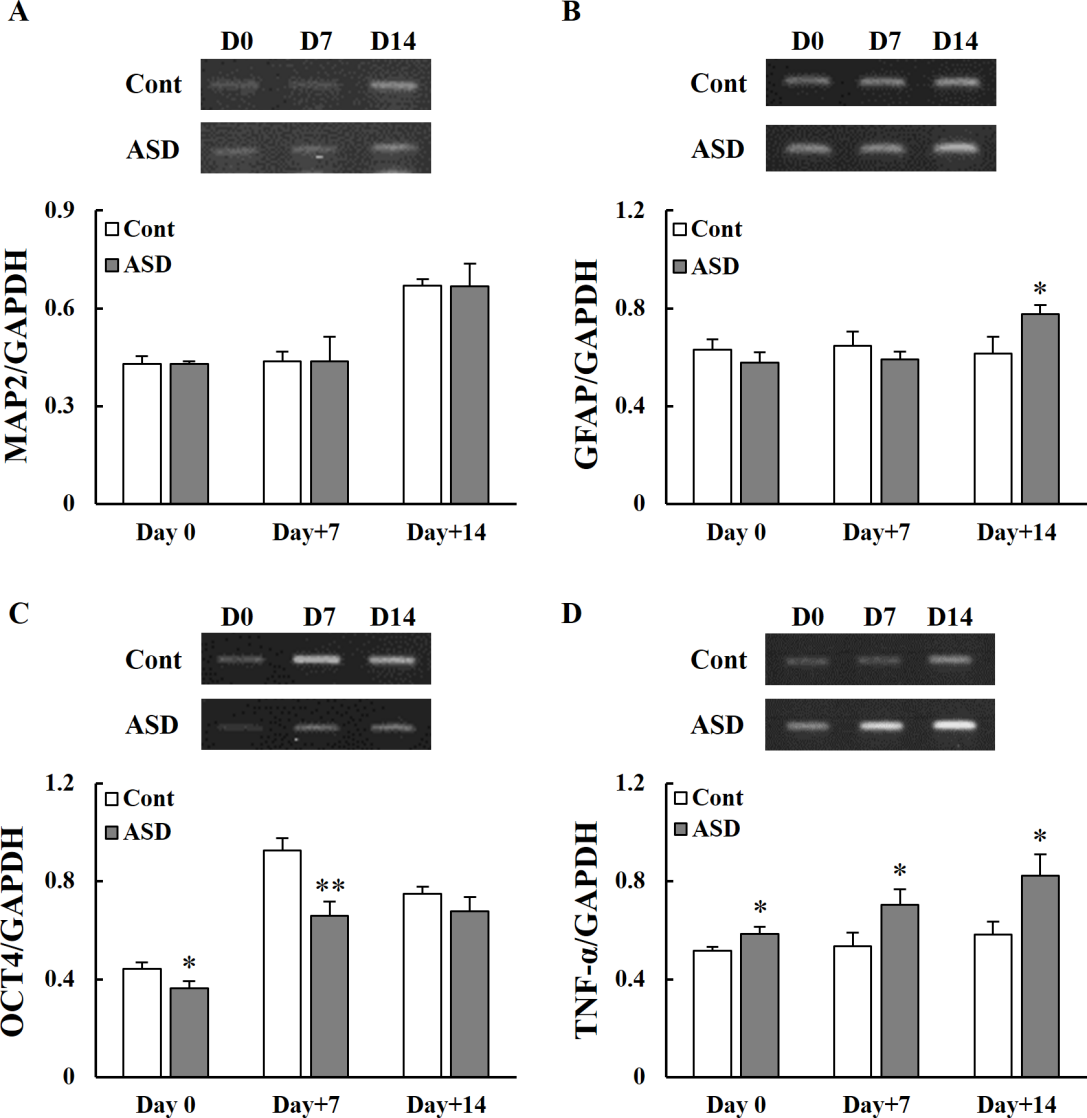
Changes in gene expression in the hippocampus between early and the late post- induction day following PPA administration. Gene expression of a neuron-specific microtubule associated protein (*MAP2*) (A), an astrocyte marker (*GFAP*) (B), a neural stem cell marker (*OCT4*) (C), and a pro-inflammatory cytokine (*TNF-α*) (D) following PID 0 and during the late PID period (days 7 and 14). Data are shown as the mean ± SD. Cont, control rats; ASD, PPA-treated rats. ^*^p < 0.05, ^**^p < 0.01: *vs*. Cont.

Gene expression in the hippocampus of 3-week-old rats following PPA administration was analyzed by reverse transcription PCR. The expression of microtubule-associated protein 2 (*MAP2*), glial fibrillary acidic protein (*GFAP*), octamer-binding transcription factor 4 (*OCT4*), and *TNF-α* was measured on PID 0, 7 and 14 in control and PPA-treated rats. The expression of *MAP2*, a neuron-specific microtubule-related gene, was not significantly altered between the control and PPA-treated rats (*p < 0.05; Fig 5A). The expression of *GFAP*, an astrocyte-related gene, was significantly increased on PID 14 in the PPA-treated rats (*p < 0.05; Fig 5B). The expression of *OCT4*, a neural stem cell-related gene, was significantly decreased on PID 0 and 7 in PPA-treated rats (*p < 0.05 and **p < 0.01, respectively; Fig 5C). In addition, the PPA-treated rats showed significantly increased expression of the pro-inflammatory gene *TNF-α* compared with the control group (*p < 0.05; Fig 5D).

### Histological alterations of the hippocampus following PPA administration

**Fig 6:**
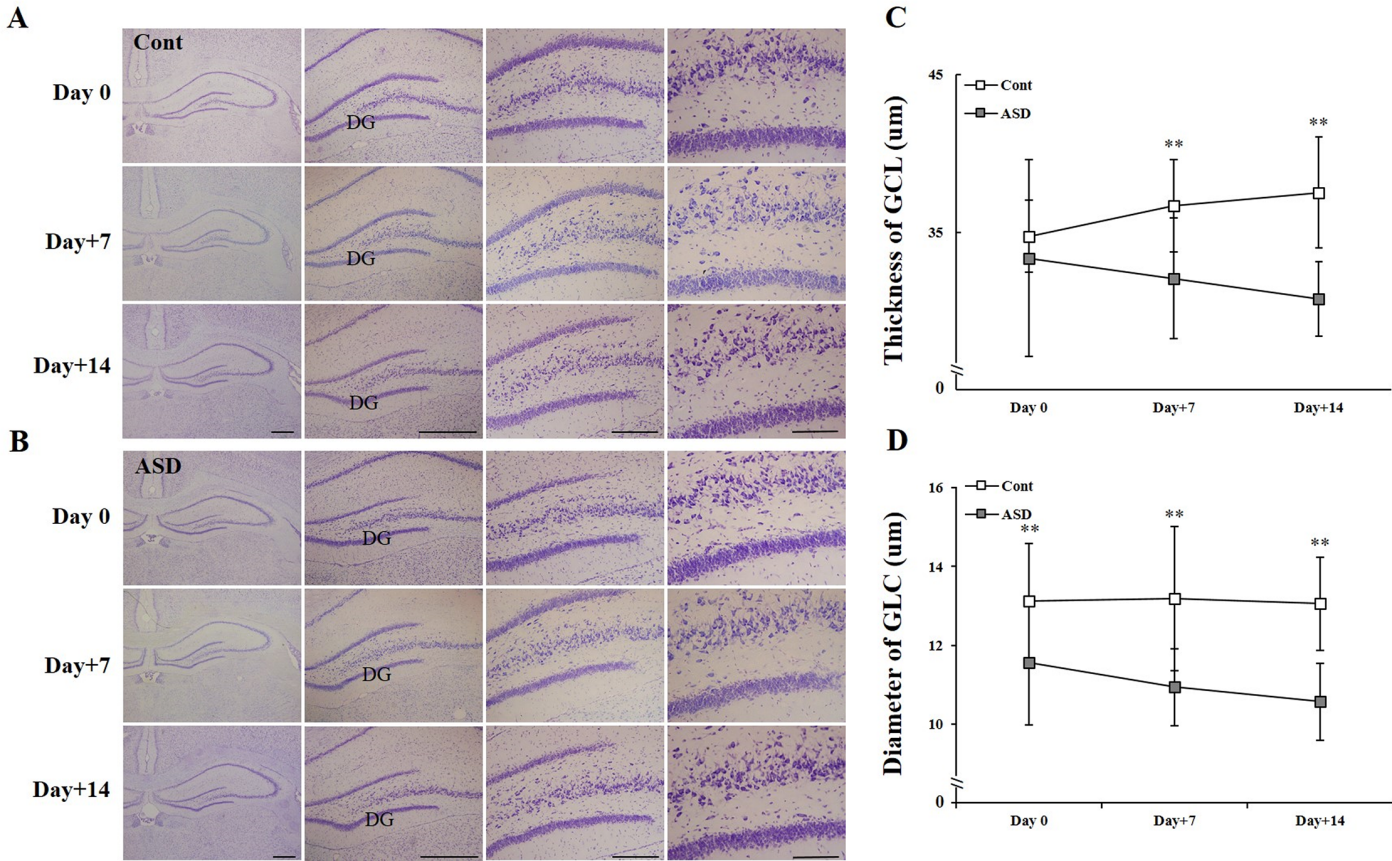
Structural changes of hippocampal tissue in the control and PPA-treated rats. Histological changes in the dentate gyrus of the hippocampus between the early and late PID period following PPA administration (A, B). Quantitative results for GCL thickness (C) and GC diameter (D). Data are shown as the mean ± SD. Cont, control rats; ASD, PPA-treated rats; GCL, granule cell layer; GC, granule cell. ^**^p < 0.01: *vs*. Cont.

To evaluate the structural changes in the dentate gyrus of hippocampus following PPA administration, we performed Nissl staining in both control and PPA-treated rats (Fig 6A and 6B). Furthermore, we measured the thickness of the dentate gyrus of the hippocampus between PID 0 and the late PID period (days 7 and 14) following PPA treatment. Control rats showed increased GCL thickness. However, PPA-treated rats exhibited a significantly reduced GCL thickness compared with control rats on PID 7 and 14 (**p < 0.01; Fig 6C). Regarding the granule cell diameter, PPA-treated rats showed a significant reduction during whole PID period (days 0, 7 and 14) (**p < 0.01; Fig 6D).

### GFAP immunostaining

**Fig 7:**
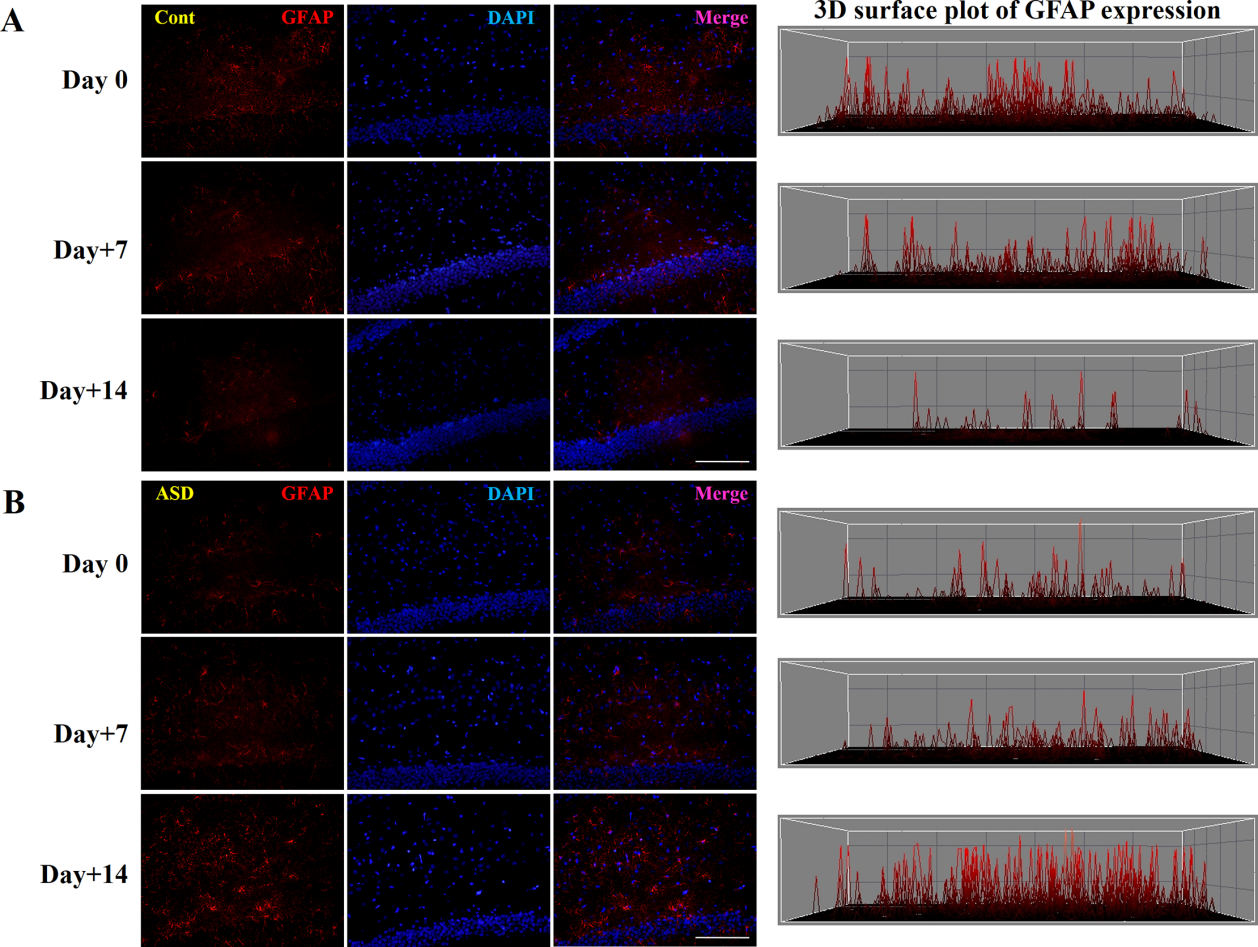
Immunostaining of hippocampal tissue with GFAP following PPA administration during early and late post-induction day. GFAP-positive cells (red) in the hippocampus in control (A) and PPA-treated (B) rats. To quantify the number of GFAP-positive cells, we obtained a 3D surface plot using Image-Pro Plus software. Cont, control rats; ASD, PPA-treated rats. Scale bar = 200 μm, *p* < 0.01: *vs*. Cont.

To evaluate histological changes in the hippocampus following PPA administration, GFAP, a marker of astrocyte expression in the hippocampus, was analyzed (Fig 5B). The level of GFAP-positive cells was increased in the PPA-treated compared with the control rats on PID 14 (Fig 7). In addition, there was a significant difference in levels between PID 0 and 14 (*p* < 0.01; Fig 7B; only a three-dimensional [3D] surface plot is shown)

## Discussion

The optimal PPA dose acts as a precursor for glucose production to make energy in ruminants and affects human physiological actions. Since some foods such as wheat and dairy products contain PPA as a preservative, such food intake may exacerbate the symptoms of ASD [20]. In human, PPA is generated through the fermentation of polysaccharides and oligosaccharides by propionibacteria including *bacteroidetes* flora, *bacteroides*, *desulfovibrio*, and other bacteria (*clostridials*) [33–36]. However, feces from autistic patients have an altered microfloral profile, including alterations in *bacteroidetes* and *firmicutes*, compared with those from normal subjects [34]. Recently, several studies have reported that the use of antibiotics, which lead to changes in microflora, affects the regulation of the gut-brain axis and results in neurobehavioral deficits, as well as metabolic and psychiatric disorders, such as anxiety and memory loss [37–39]. The microbiota is largely influenced by foods. A few foods, such as Swiss cheese, include PPA, which is also used for weight loss. Large amounts of PPA are used in agriculture and industry [34].

PPA injection triggers ASD-like behaviors and neuroinflammatory reactions in animals. Like other mammals, the brains and bodies of neonatal rats develop in concert with motor skills and behaviors during the first 3 weeks of life. During that time, the injection of PPA acts like a neurotoxic agent, and rats exhibit abnormal behavioral patterns, such as abnormal social interactions and anxiety-like behavior. Their brains exhibit inflammation and abnormal neurotransmission and oxidative stress induced by inhibition of Na^+^/K^+^ ATPase and increased sensitivity of the glutamate receptor [40–42]. PPA may accumulate within certain types of cells, triggering acidification and thus affecting neurotransmitter synthesis and release. PPA can cross the blood-brain barrier to reach the CNS [19]. PPA induces mitochondrial diseases via environmental factors [36], which are associated with GI disorders and directly affect GI symptoms. In addition, the administration of PPA affects gut motility and smooth muscle contraction. ASD patients exhibit GI symptoms, and MacFabe [36] reported that oxidative stress and abnormal GSH levels were increased in brain tissue, while the activities of antioxidant enzymes, including superoxide dismutase, catalase, glutathione peroxidase, and GSH reductase, were reduced in PPA-treated animal models. PPA expression via transient transfection can epigenetically modulate PC12 cell function [23], and in autistic lymphoblastoid cell lines, PPA treatment resulted in loss of mitochondrial function in a concentration- and time-dependent manners [43]. Additionally, Frye et al. [43] reported that these changes were related to increased reactive oxygen species levels and proton leakage. Therefore, we investigated the effects of PPA on the development of ASD, hippocampal dentate gyrus morphology, and neurobehavior in rat pups.

In this study, we produced an autism-like animal model using PPA treatment, and measured time-dependent neurobehaviors, changes in gene expression, and histological alterations. PPA-treated rats exhibited significantly reduced body weight during the late PID period of ASD (Fig 2). Previous studies reported that short chain fatty acids, such as PPA, affect weight loss in both animal and human subjects [44, 45]. Additionally, PPA acts as a gluconeogenic substrate, and is involved in gluconeogenesis upon entry into the citric acid cycle [46]. Another possible reason of weight loss in PPA-treated rats is related to abnormal fatty acid metabolism [23].

Previous studies reported that ASD rats exhibit autism-like symptoms, including impaired cognition and restricted-repetitive behaviors, in areas of interest to them [14, 20, 21, 25, 26, 47, 48]. PPA-treated rats also displayed time-dependent attenuation of exploratory activity as well as restricted locomotor activity in outer squares that represent an unfamiliar environment (Fig 3). Shultz et al. [21] reported that intracerebroventricular PPA administration increased repetitive behaviors, movement, and turning behavior. Our analysis of social behavior revealed that PPA-treated rats showed aggressive social behaviors, including following and chasing, anogenital interaction, and head-to-head interaction, and presented with excessively aggressive behavior (Fig 4). These neurobehavioral changes may be caused by PPA administration and resultant CNS alterations, not only by peripheral irritation. In this study, each cage housed two rats from the control or PPA-treated rats. Although this housing method would induce the aggressive behaviors itself, however, the important thing to note was the appearance of altered behavioral patterns following PPA administration rather than the extent of altered neurobehaviors. Additionally, aggressive behavior known as peripheral irritant effects may be generated by the injection of buffered PPA. To determine the effect of buffered PPA injection on production of ASD in an animal model, we performed reverse transcription PCR and histologic analyses.

In addition, anxiety-like behavior, a representative symptom of ASD, may be generated through decreased levels of neurotransmitter via downregulated gamma-amino butylic acid (GABA) synthesis and upregulated GABA transporter activity in ASD-like rats [49]. We investigated the expression of *MAP2*, *GFAP*, and *OCT4*. The expression of *MAP2* was similar between the two groups. However, the expression of *GFAP* was significantly reduced on PID 14 in PPA-treated rats compared with control rats. The expression of *OCT4* was also significantly decreased on PID 0 and 7 in PPA-treated rats (Fig 5). Our results also revealed increased expression of the TNF-α in the hippocampus of the PPA-treated rats. These results are related to reduction of neural plasticity or cell size and increased neuronal loss. MacFabe [36] revealed reactive astrogliosis and microglial activation but found no significant difference in caspase 3 expression in the hippocampus. MacFabe et al. [15] reported that PPA administration was associated with increased expression of *GFAP* and may induce astrogliosis, which is consistent with our study.

Similarly, a previous study suggested that autism-like behavior may be induced by overexpression of pro-inflammatory cytokines, such as IL-6, which regulate the organization of neural cells in the brain [50]. Excessive pro-inflammatory cytokine levels may be associated with neural cell differentiation and maturation during development [51]. Wei et al. [50] suggested that IL-6 is an important mediator of autism-like behaviors. When IL-6 was overexpressed, neural circuit imbalances and abnormalities in synaptic plasticity were evident, followed by impaired cognition, learning deficits, abnormal anxiety-related behavior, and reduced social interactions. In this process, increased pro-inflammatory cytokines were implicated in neural cell death via activation of apoptosis. In a future study, we will focus on identifying neuronal cell characteristics, such as organization of hippocampal excitatory and inhibitory neurons, after PPA treatment.

Humans and animals with ASD exhibit altered functions and structures in various brain areas, such as the cortex (i.e., prefrontal cortex [52], anterior cingulate cortex, insular cortex [53]), and hippocampus [8, 15, 36, 54–56]. Likewise, an increased number of neurons and altered density and connectivity of the dendritic spine have been reported in humans and animals with ASD [55]. Additionally, altered levels of various molecules including NMDA, AMPA, Shank, MECP, and PTEN, in global brain regions have been reported [57, 58]. In our histological analysis, PPA-treated rats showed a time-dependent reduction in GCL thickness and GC diameter (Fig 6). Saitoh et al. [59] reported smaller cross-sectional areas of the dentate gyrus and the Cornu Ammonis (CA) region 4 in patients with ASD than in normal subjects, with the most remarkable differences evident in those aged 2–4 years. Thus, disrupted neurodevelopment could promote disturbances in the differentiation and maturation of hippocampal neural cells. In addition, the increased number of astrocytes may lead to astrogliosis and abnormal synaptogenesis (Fig 7). Consequently, the hippocampi of adolescent rats may be organizationally immature or suffer from arrested neurodevelopment and physiological programmed cell death, such as apoptosis [60].

Patients with ASD may be affected by mitochondrial disease. Common biomarkers have been discovered between patients with ASD and those with mitochondrial disease. In particular, the brain of PPA-induced ASD-like animal models and patients with ASD revealed augmented long-chain acylcarnitines caused by abnormal fatty acid metabolism, which has been associated with loss of mitochondrial function via the tricarboxylic-acid cycle [43, 48, 61]. Additionally, an abnormal pattern of acylcarnitine levels may affect glutathione metabolism in PPA-induced ASD animals [61]. To investigate the mechanism between PPA injection and ASD, altered cells in the CNS should be confirmed. In addition, pyrosequencing of microbes is needed to elucidate the potential correlation between PPA injection and propionibacteria. In this study, we obtained some interesting findings related to neurobehavioral deficits, including social interactions in ASD-like animal models [20]. We revealed that psychologic, physiologic, and histologic changes are induced by PPA administration in terms of mitochondrial dysfunction, abnormal acylcarnitine levels due to impaired fatty acid metabolism, and altered glutathione metabolism.

Therefore, we conclude that daily PPA administration for 5 consecutive days may modulate certain pathological changes and contribute to the development of autism. One previous study reported that PPA was detected in blood samples up to 60 min after its subcutaneous injection [62]. In this study, a rat ASD model was developed by subcutaneous PPA administration at a daily dose of 500 mg/kg (250 mg/mL, 0.26 M, pH 7.4) for 5 consecutive days. We showed altered gene expression, abnormal behaviors, and histological changes. Therefore, we suggest a modified method for production of an ASD animal model.

Crawley [63] insisted that the ideal autism animal model must have at least three diagnostic symptoms characteristic of clinical patients, including abnormal social interactions deficits in social communication. Our study performed to expand the field by utilizing ASD-like animal models by PPA administration. We hope that the animal model with high validity established in this study is expected to clarify the diet-related mechanisms, gastrointestinal issues, microbiota, and metabolites in patients with ASD.

In conclusion, autism is a severe neurodevelopmental disorder characterized by impaired social interactions, deficits in verbal and non-verbal communication, repetitive behavior, and restricted interests. However, the etiology of the disorder remains poorly understood. This study suggests that PPA administration may induce critical changes involving abnormal neural cell organization followed by autism-like neurobehaviors, including increased aggressive behavior, reduced exploratory activity, and isolative and passive behavior.

## Supporting information

S1 Data Raw data of all figures in this paper. (XLSX)

S1 Fig Food consumption in control and PPA-treated animals. Food consumption were measured in control and PPA-treated groups. There were no significant difference between control and PPA-treated group. Data are presented as mean±SD. Cont, control rats; ASD, PPA-treated rats.
(DOCX)

S1 Video Video tracking system for monitoring social behavior tests. To verify the abnormal social behaviors in PPA-treated animals, video tracking system was used.
(EGG)
