# Supplementary material for: Pathophysiological and neurobehavioral characteristics of a propionic acid-mediated autism-like rat model
Source: PLoS One. 2018 Feb 15;13(2):e0192925. doi: 10.1371/journal.pone.0192925 (PMC5814017; doi:10.1371/journal.pone.0192925)
Supplement: S1 Fig — Food consumption were measured in control and PPA-treated groups. There were no significant difference between control and PPA-treated group. Data are presented as mean±SD. Cont, control rats; ASD, PPA-treated rats. (DOCX) [file pone.0192925.s002.docx]

**S1 Figure**

**Food consumption in control and ASD animals.** Food consumption were measured in control and ASD groups. There were no significant difference between control and ASD group. Data are presented as mean±SD. Cont: control rats, ASD: PPA-induced autism-like rats.


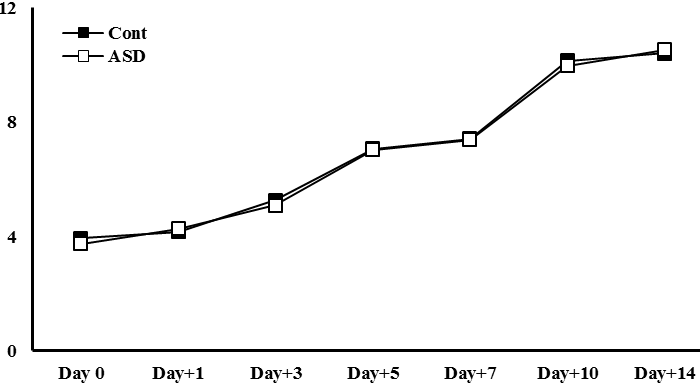


**Food consumption (g)**
